# Lower testosterone levels predict increasing severity and worse outcomes of hepatitis B virus-related acute-on-chronic liver failure in males

**DOI:** 10.1186/s12876-021-01993-1

**Published:** 2021-12-06

**Authors:** Yandi Huang, Dong Yan, Huafen Zhang, Bin Lou, Ren Yan, Yifan Yao, Minya Dong, Donglei Yang, Feifei Lv, Yu Chen

**Affiliations:** 1 Department of Laboratory Medicine, the First Affiliated Hospital, College of Medicine, Zhejiang University, #79 Qingchun Road, Hangzhou, 310000 Zhejiang People’s Republic of China; 2 Key Laboratory of Clinical In Vitro Diagnostic Techniques of Zhejiang Province, Hangzhou, 310000 China; 3 Institute of Laboratory Medicine, Zhejiang University, Hangzhou, 310000 China; 4 State Key Laboratory for Diagnosis and Treatment of Infectious Diseases, Collaborative Innovation Center for Diagnosis and Treatment of Infectious Diseases, the First Affiliated Hospital, College of Medicine, Zhejiang University, Hangzhou, 310000 China

**Keywords:** Hepatitis B, Acute-on-chronic liver failure, Testosterone, Androgen

## Abstract

**Background:**

Hepatitis B virus-related acute-on-chronic liver failure (HBV-ACLF) is a serious liver disease with pathogenesis remaining unclear. This study aims to investigate the association between testosterone levels, stage (early, middle, or late, categorized according to clinical manifestation), severity scores, and clinical outcomes of HBV-ACLF.

**Methods:**

This single-center observational study involved 160 male patients with HBV-ACLF, 151 chronic hepatitis B patients without liver failure (CHB) and 106 healthy controls (HC). Morning blood samples were collected and androgen levels analyzed by chemi-bioluminescent immunoassay. Time to death or liver transplantation within 90 days comprised the primary composite outcome.

**Results:**

Serum levels of total testosterone (TT), free testosterone index (FTI), dehydroepiandrosterone sulfate and cortisol were significantly lower among HBV-ACLF than CHB and HC, while androstenedione was higher. Low TT, sex hormone binding globulin and FTI were associated with increased stage (of HBV-ACLF, ascites, and hepatic encephalopathy) and severity scores (Model for End-stage Liver Disease and Chinese Group on the Study of Severe Hepatitis B-ACLF scores). Low TT (< 142.39 ng/dL) was a risk factor for both the composite outcome and for death alone within 90 days. Multivariate analysis revealed TT to be an independent predictor for the composite outcome (hazard ratio 2.57, 95% CI 1.09–6.02; *P* = 0.030).

**Conclusion:**

Low serum testosterone is common among male patients with HBV-ACLF and predictive of increased severity and worse outcome of the disease and may play an important role in the progression of HBV-ACLF.

## Background

Acute-on-chronic liver failure (ACLF) is a complex syndrome, characterized by acute and severe liver injury in the context of pre-existing chronic liver disease [1]. Among Asian populations, the major underlying disease for ACLF is hepatitis B virus (HBV) infection [2]. Hepatitis B virus-related acute-on-chronic liver failure (HBV-ACLF) is associated with a high rate of short-term mortality, ranging from 58 to 74% [3], while the 5-year survival rate of patients with HBV-ACLF has been reported to be up to 97.2% for those who survive beyond 90 days after diagnosis [4]. Unfortunately, the lack of understanding of HBV-ACLF pathogenesis prevents a rapid identification and effective treatment to reduce the mortality of the disease. Recent reports have revealed immune-metabolism disorder during the development of HBV-ACLF by both peripheral blood mononuclear cells transcriptomics analysis [5] and circulating proteomic panels analysis [6].

Testosterone, the most important androgen in males, plays a critical role in the metabolism of proteins, carbohydrates, and fat; and is believed to have suppressive effects on the immune system [7]. And its deficiency may contribute to increasing fat, decreasing muscle mass [8], intense immune response to HBV infection and severe liver inflammation [9]. Recent data have demonstrated that, testosterone deficiency is of high incidence among men with end-stage liver disease; furthermore, low testosterone is associated with an increased risk of mortality regardless of the etiology of disease [10–12]. Xu et al. [13] found that serum testosterone was decreased in acute liver failure compared with in hepatitis B flare. However, there have been no clinical trials to date exploring the relationship between testosterone levels and the state of HBV-ACLF.

Considering the dynamic transformation between the bound and free forms of testosterone, interactions between steroid hormones and the potential influences of sex, age, body mass index (BMI), and other clinical factors, we sought to compare the circulating serum levels of total testosterone (TT), free testosterone index (FTI), sex hormone binding globulin (SHBG), dehydroepiandrosterone sulfate (DHEAS), cortisol and androstenedione (AND) in men with HBV-ACLF with those of age- and BMI-matched chronic hepatitis B patients and healthy controls without liver disease. Secondarily, we evaluated whether the five androgens and SHBG are associated with the severity and outcome of HBV-ACLF in cases. We hypothesized that low testosterone is related to increased severity of disease and risk of death or liver transplantation among men with HBV-ACLF. Deeper understanding of the role and functions of testosterone levels in HBV-ACLF may contribute to further studies to identify contributors to severity and mortality of liver disease and determine if application of testosterone therapy improves patient outcomes.

## Methods

### Study design and overview

This is a single-center, prospective observational study. The study were performed in accordance with the ethical guidelines of the 2013 Declaration of Helsinki and all its protocols were approved by the Clinical Research Ethics Committee of the First Affiliated Hospital, Zhejiang University School of Medicine (approval number 2017[674]). Written informed consent was obtained from all the participants and for dead participants from their legal representatives prior to enrollment in the study.

### Subjects

We consecutively recruited 229 patients with HBV-ACLF who underwent treatment with an artificial liver support system at the First Affiliated Hospital of Zhejiang University College of Medicine during the 14-month period between December 2018 and January 2020 by invitation. Of these, 69 were excluded from analysis (30 who were female, 28 who had other chronic liver disease, five with hepatocellular carcinoma, four who had undergone previous liver transplant, one who had undergone stem-cell transplantation, and one with pituitary tumor). During the study period, we recruited 151 age- and BMI-matched males with chronic hepatitis B but without liver failure from sample bank of major diseases in Key Laboratory of Clinical In Vitro Diagnostic Techniques of Zhejiang Province as disease controls and 106 matched healthy controls without liver disease from the physical examination center. Exclusion criteria were: other diagnosed chronic liver disease (such as alcoholic liver disease, autoimmune liver disease, drug-induced liver injury, or other viral infection); hepatocellular carcinoma; previous transplant; hypogonadism; the presence of testicular, prostatic, or pituitary tumor; and use of hormone therapy (including estrogen, progesterone, testosterone compounds, or dehydroepiandrosterone supplements) or anticoagulation therapy such as warfarin.

### Clinical diagnosis

The diagnostic criteria for HBV-ACLF followed the criteria of the Chinese Group on the Study of Severe Hepatitis B (COSSH) [3]: acute hepatic insult manifesting severe jaundice (total bilirubin [Tbil] ≥ 205 μmol/L) and coagulopathy (international normalized ratio [INR] ≥ 1.5) develops in patients with CHB and regardless of the presence of cirrhosis. We categorized subjects according to stage of HBV-ACLF; namely, early stage (1.5 ≤ INR < 1.9 without complications or extrahepatic organ failure), middle stage (1.9 ≤ INR < 2.6 with one complication and/or one extrahepatic organ failure), or end stage (INR ≥ 2.6 with two or more complications and/or extrahepatic organ failures).

### Data collection

Clinical data was obtained from medical records relating to essential information (age, gender, weight, height), comorbidities (diabetes mellitus, hypertension), precipitating events, laboratory indexes (such as serum alanine aminotransferase [ALT], aspartate aminotransferase [AST], albumin, Tbil, INR, creatinine level, complete blood count), and complications (ascites, hepatic encephalopathy, infection, acute kidney injury, gastrointestinal hemorrhage). The Model for End-stage Liver Disease (MELD) [14] score was calculated using the following formula: (9.6 × LogE_creatinine [mg/dL]_) + (3.8 × LogE_Tbil [mg/dL]_) + (11.2 × LogE_INR_) + 6.4 and COSSH-ACLF [3] score: 0.741 × INR + 0.523 × HBV-SOFA (the sum of the scores for four organ systems [renal, hepatic, circulatory, and respiratory systems]) + 0.026 × age + 0.003 × Tbil. Both the two scores were calculated based on the baseline variables. The primary outcome, defined as death or transplantation within 90 days of first treatment of artificial liver support system, was analyzed through evaluation of medical records or by direct contact with the subjects or their families.

### Laboratory analysis

Morning fasting blood samples were drawn from all subjects prior to treatment with the artificial liver support system and from controls at the time of recruitment. We analyzed the androgens using chemi-bioluminescent immunoassay. Serum TT, SHBG and DHEAS were measured on an Architect i4000 analyzer (Abbott Laboratories, Kallang Place, Singapore), cortisol was measured on an ADVIA Centaur XP (Siemens Healthcare Diagnostics Inc., Los Angeles, CA, USA) and androstenedione was measured on an Immulite 2000XPi (Siemens Healthcare Diagnostics Inc.). The FTI was calculated for each participant as (TT × 10)/SHBG [26]. The normal ranges for TT, SHBG, FTI, DHEAS, cortisol and androstenedione in males are 142.39–923.14 ng/dL, 17.1–77.6 nmol/L, 20.4–81.2%, 48.6–591.9 μg/dL, 5.27–22.45 μg/dL and 0.6–3.1 ng/mL, respectively.

### Statistical analysis

All continuous variables are expressed as median, interquartile range (IQR; 25th and 75th percentiles), categorical data are presented as percentage and frequency. TT, FTI and SHBG levels were natural log-transformed and other androgen levels were square root-transformed. In univariate statistical comparisons, the Mann–Whitney non-parametric U test was used for continuous variables and the Kruskal–Wallis test to compare more than two groups. Categorical data were evaluated using a chi-squared test or Fisher’s exact test, as appropriate. Generalized linear models were used to predict an increase in stage of HBV-ACLF, ascites, and hepatic encephalopathy with decreasing or increasing androgen level. The relationships between androgen level and severity scores were examined in subjects with HBV-ACLF through multiple linear regression analysis. Kaplan–Meier estimation was used to evaluate the survival rates without transplant of groups with different testosterone levels. The log-rank test was used to compare mortality rates in terms of the composite outcome and in terms of death alone between groups with different testosterone levels. When analyzing mortality rates, subjects who underwent liver transplant were excluded. With regards to HBV-ACLF prognosis, the Cox proportional hazards model was fitted with a forward stepwise selection method (p-in: 0.05 and p-out: 0.10) to identify risk factors associated with the composite outcome of death or transplantation. All statistical analyses were carried out using SPSS version 24.0 (IBM Corp., Armonk, NY). A value of *P* < 0.05 was considered statistically significant.

## Results

### Characteristics of the study population

A total of 160 males aged 45 (36–56) years with HBV-ACLF were enrolled for analysis in the present study. Of these, 32 were categorized as having early stage liver failure, 61 as middle stage, and 67 as end stage. The most frequent potential precipitating event was hepatitis B relapse (58.1%), followed by bacterial infection (18.8%), hepatotoxic drugs (3.7%), superimposed HEV infection (1.3%), gastrointestinal hemorrhage (1.3%), more than one hepatic insult (5.6%) and unknown cause (11.2%). All patients received antiviral therapy upon diagnosis of hepatitis B and patients with HBV-ACLF underwent plasma exchange every 1 or 2 days until improvement or the primary outcome. All 160 subjects completed follow-up. The in-hospital 90-day mortality rate was 34.4% (21 subjects underwent liver transplantation and 34 died).

Table 1 compares various characteristics between subjects, chronic hepatitis B patients and healthy controls, as well as in relation to stage of ACLF. Age, BMI, and incidence of basic diseases including diabetes and hypertension were not significantly different between groups. Laboratory data (except ALT and AST) were similar in chronic hepatitis B patients and healthy controls, while TT, FTI, DHEAS and cortisol levels were significantly lower in subjects than chronic hepatitis B patients and healthy controls, and androstenedione was higher. When HBV-ACLF patients with cirrhosis (82 patients) were excluded from subjects, the performance of androgens was consistent. According to the lower limit of the reference range of TT for males, 65% of subjects were TT deficient and 85.6% were FTI deficient. The rate of TT deficiency was highest among end-stage subjects while FTI deficiency was more common among middle-stage subjects compared with other stages. Increased stage of HBV-ACLF was associated with higher rates of ascites, hepatic encephalopathy, and infection. The rates of acute kidney injury and gastrointestinal hemorrhage were very low in our cohort. The MELD and COSSH-ACLF scores and short-term mortality rate increased with increased stage of HBV-ACLF.

**Table 1:** Clinical characteristics of the study population

Characteristic	Healthy controls (n = 106)	Chronic Hepatitis B (n = 151)	HBV-ACLF (n = 160)	HBV-ACLF (n = 160)		
				Early stage (n = 32)	Middle stage (n = 61)	End stage (n = 67)
Age (years)	43 (35–52)	43 (35–51)	45 (36–56)	40 (32–56)	49 (39–57)	45 (37–53)
BMI (kg/m^2^)	23.3 (21.6–25.2)	23.4 (21.8–26.6)	24.0 (21.8–26.3)	24.0 (21.3–26.5)	23.9 (21.8–25.7)	24.2 (21.6–26.4)
Diabetes, % (n)	7.5% (8)	7.3% (11)	8.1% (13)	3.1% (1)	11.5% (7)	7.5% (5)
Hypertension, % (n)	17.0% (18)	14.6% (22)	18.1% (29)	12.5% (4)	23.0% (14)	16.4% (11)
ALT (U/L)	15 (12–21)	27 (19–35)^a^	234 (116–442)^b^	305 (182–466)	158 (101–274)^c^	269 (112–587)^d^
AST (U/L)	19 (16–22)	24 (19–32)^a^	129 (84–229)^b^	158 (78–350)	110 (75–147)^c^	156 (92–280)^d^
Albumin (g/dL)	47 (45–49)	48 (46–51)	31 (29–34)^b^	33 (31–36)	31 (29–33)^c^	31 (28–34)
Tbil (μmol/L)	11 (9–13)	13 (10–18)	350 (285–431)^b^	304 (264–421)	369 (290–420)	370 (305–486)
Platelet count (10^9^/L)	222 (192–264)	193 (161–230)	106 (71–138)^b^	139 (108–193)	100 (73–127)^c^	98 (61–128)
TT (ng/dL)	567 (444–714)	573 (444–743)	101 (61–202)^b^	291 (129–481)	107 (59–187)^c^	76 (45–130)
SHBG (nmol/L)	33 (27–47)	37 (28–88)	40 (28–55)	55 (43–75)	47 (31–57)	32 (22–43)^d^
FTI (%)	57.3 (46.3–68.3)	53 (41–64)	9.7 (6.1–15.5)^b^	17.6 (9.8–25.8)	8.4 (6.4–11.9)^c^	8.1 (5.4–13.7)
DHEAS (μg/dL)	304 (232–380)	286 (183–364)	152 (86–283)^b^	183 (109–394)	106 (48–200)^c^	194 (97–292)^d^
Cortisol (μg/dL)	13.2 (9.3–16.0)	11.7 (8.9–15.7)	9.1 (6.9–12.5)^b^	9.4 (7.5–14.8)	8.8 (6.5–11.6)	11.1 (5.5–15.0)
Androstenedione (ng/mL)	1.7 (1.3–2.3)	1.8 (1.4–2.1)	3.5 (2.3–4.7)^b^	3.5 (2.9–4.8)	2.8 (1.6–3.7)	4.2 (2.6–5.6)^d^

*HBV-ACLF* Hepatitis B virus-related acute-on-chronic liver failure, *BMI* body mass index, *ALT* alanine aminotransferase, *AST* aspartate aminotransferase, *Tbil* total bilirubin, *TT* total testosterone, *SHBG* sex-hormone-binding globulin, *FTI* free testosterone index, *DHEAS* dehydroepiandrosterone sulfate

Data are expressed as median (interquartile range; 25th and 75th percentiles) or percentage (frequency). *P* value < 0.05 for comparisons between chronic hepatitis B and healthy controls^a^, HBV-ACLF and chronic hepatitis B^b^, middle stage and early stage^c^, and between end stage and middle stage^d^

### Association of androgen levels with severity of hepatitis B virus-related acute-on-chronic liver failure

Lower levels of TT, FTI, and SHBG and higher levels of androstenedione were associated with increased risk of advanced severity stage (Table 2). For every unit change in TT, FTI, SHBG and androstenedione, the risk of advanced HBV-ACLF stage increased by two- to eightfold after adjustment for age and BMI. Decreased testosterone level was found to be a predictor of increased severity of ascites and hepatic encephalopathy, but FTI did not predict hepatic encephalopathy. While the prediction of increased severity of ascites with DHEAS, cortisol and androstenedione levels were weak. In addition, linear correlations were identified between androgen levels and severity scores (MELD and COSSH-ACLF scores) after adjustment for age and BMI (Table 3). For instance, each Ln (1 ng/dL) decrease in TT was associated with a higher MELD score (2.794, 95% confidence interval [CI] 1.740–3.848; *P* < 0.001).

**Table 2:** Prediction of increased stage of HBV-ACLF, ascites, and hepatic encephalopathy with decreased androgen levels

	HBV-ACLF		Ascites		Hepatic encephalopathy	
	Odds ratio (95% CI)	*P* value	Odds ratio (95% CI)	*P* value	Odds ratio (95% CI)	*P* value
1–Ln (TT [ng/dL])	3.507 (2.285–5.383)	< 0.001	2.962 (1.957–4.482)	< 0.001	3.052 (1.690–5.513)	< 0.001
1–Ln (SHBG [nmol/L])	8.757 (4.046–18.951)	< 0.001	4.293 (2.118–8.701)	< 0.001	17.010 (5.361–53.975)	< 0.001
1–Ln (FTI [%])	2.376 (1.469–3.841)	< 0.001	2.374 (1.472–3.829)	< 0.001	1.453 (0.783–2.696)	0.236
SQRT (DHEAS [μg/dL])	1.042 (0.981–1.106)	0.186	0.971 (0.915–1.031)	0.341	1.172 (1.079–1.273)	< 0.001
SQRT (cortisol [μg/dL])	1.077 (0.832–1.393)	0.573	1.028 (0.804–1.313)	0.827	1.248 (0.924–1.686)	0.148
SQRT (AND [ng/mL])	2.089 (1.139–3.830)	0.017	1.390 (0.773–2.501)	0.272	3.296 (1.473–7.375)	0.004

Model was adjusted for age and body mass index

Subjects were categorized according to stage of hepatitis B virus-related acute-on-chronic liver failure (early stage, middle stage, or end stage), ascites (none, mild ascites, or severe ascites), and hepatic encephalopathy (none, grade I/II, or grade III/IV)

*HBV-ACLF* Hepatitis B virus-related acute-on-chronic liver failure, *TT* total testosterone, *SHBG* sex-hormone-binding globulin, *FTI* free testosterone index, *SQRT* Square Root, *DHEAS* dehydroepiandrosterone sulfate, *AND* androstenedione

**Table 3:** Relationships between androgen level and severity scores in hepatitis B virus-related acute-on-chronic liver failure

	Estimate (95% CI)	*P* value
*MELD*		
1–Ln (total testosterone [ng/dL])	2.794 (1.740–3.848)	< 0.001
1–Ln (sex-hormone-binding globulin [nmol/L])	4.621 (2.727–6.515)	< 0.001
1–Ln (free testosterone index [%])	2.107 (0.703–3.512)	0.004
SQRT (dehydroepiandrosterone sulfate [μg/dL])	0.324 (0.145–0.503)	< 0.001
SQRT (cortisol [μg/dL])	1.717 (0.975–2.459)	< 0.001
SQRT (androstenedione [ng/mL])	4.982 (3.304–6.660)	< 0.001
*COSSH-ACLF*		
1–Ln (total testosterone [ng/dL])	0.527 (0346–0.708)	< 0.001
1–Ln (sex-hormone-binding globulin [nmol/L])	1.033 (0.720–1.347)	< 0.001
1–Ln (free testosterone index [%])	0.318 (0.071–0.565)	0.012
SQRT (dehydroepiandrosterone sulfate [μg/dL])	0.048 (0.034–0.062)	< 0.001
SQRT (cortisol [μg/dL])	0.234 (0.103–0.364)	0.001
SQRT (androstenedione [ng/mL])	0.790 (0.492–1.089)	< 0.001

*MELD* Model for End-stage Liver Disease, *COSSH-ACLF* Chinese Group on the Study of Severe Hepatitis B-acute-on-chronic liver failure, *SQRT* Square Root

Multiple linear regression analysis adjusted for age and body mass index. Estimates (slopes) are given with 95% confidence intervals within parentheses

The clinical characteristics and outcomes of subgroups after stratification by TT are presented in Table 4. Low TT was found to be associated with advanced disease stage; MELD and COSSH-ACLF scores; levels of liver-function indicators, prevalence of ascites and hepatic encephalopathy, and in-hospital mortality. Infection exhibited a stepwise increase with increasing stage of HBV-ACLF but was not more frequent among subjects with low TT. There were no significant differences in age, BMI, or incidence of basic diseases between subjects with high or low TT.

**Table 4:** Clinical characteristics in relation to total testosterone level

	TT < 142.39 ng/dL (n = 104)	TT ≥ 142.39 ng/dL (n = 56)	*P* values
*Stage of acute-on-chronic liver failure, % (n)*			
Early stage	9.6% (10)	39.3% (22)	< 0.001
Middle stage	38.5% (40)	37.5% (21)	< 0.001
End stage	51.9% (54)	23.2% (13)	< 0.001
*Complications, % (n)*			
Ascites	80.8% (84)	48.2% (27)	< 0.001
Mild Ascites	49% (51)	41.1% (23)	< 0.001
Severe Ascites	31.7% (33)	7.1% (4)	< 0.001
Hepatic encephalopathy	31.7% (33)	3.6% (2)	< 0.001
Grade I or II	12.5% (13)	1.8% (1)	< 0.001
Grade III or IV	19.2% (20)	1.8% (1)	< 0.001
Infection	26.9% (28)	28.6% (16)	0.824
Acute kidney injury	7.7% (8)	0% (0)	0.051
Gastrointestinal hemorrhage	2.9% (3)	0% (0)	0.552
*Laboratory data*			
Albumin (g/dL)	31 (28–34)	32 (31–34)	0.01
Total bilirubin (μmol/L)	378 (301–468)	309 (256–398)	< 0.001
Total cholesterol (mmol/L)	2.0 (1.6–2.3)	2.5 (1.9–4.9)	< 0.001
Hemoglobin (g/L)	123 (111–133)	128 (119–135)	0.03
Platelet count (10^9^/L)	100 (67–126)	120 (91–180)	< 0.001
C-reactive protein (mg/L)	12.3 (8.2–20.1)	9.6 (6.0–16.2)	< 0.001
Sex-hormone-binding globulin (nmol/L)	32 (25–46)	54 (44–72)	< 0.001
Free testosterone index (%)	7.0 (5.3–9.7)	18.0 (12.5–24.1)	< 0.001
Dehydroepiandrosterone sulfate (μg/dL)	160 (88–310)	127 (70–218)	0.06
Cortisol (μg/dL)	9.2 (7.1–13.2)	8.9 (5.7–12.2)	0.44
Androstenedione (ng/mL)	3.35 (2.23–4.97)	3.60 (2.32–4.47)	0.69
*Severity score*			
MELD	33 (29–35)	29 (26–31)	< 0.001
COSSH-ACLF	6.7 (6.2–7.6)	5.8 (5.5–6.3)	< 0.001
*Mortality, % (n)*			
90 days	45.2% (47)	14.3% (8)	< 0.001

Data are expressed as median (interquartile range; 25th and 75th percentiles) or percentage (frequency)

The mortality refers to rate of composite outcome defined as death or transplantation

*MELD* Model for End-stage Liver Disease, *COSSH-ACLF* Chinese Group on the Study of Severe Hepatitis B-acute-on-chronic liver failure

### Association of androgen levels with the composite outcome of death or liver transplant

**Fig. 1:**
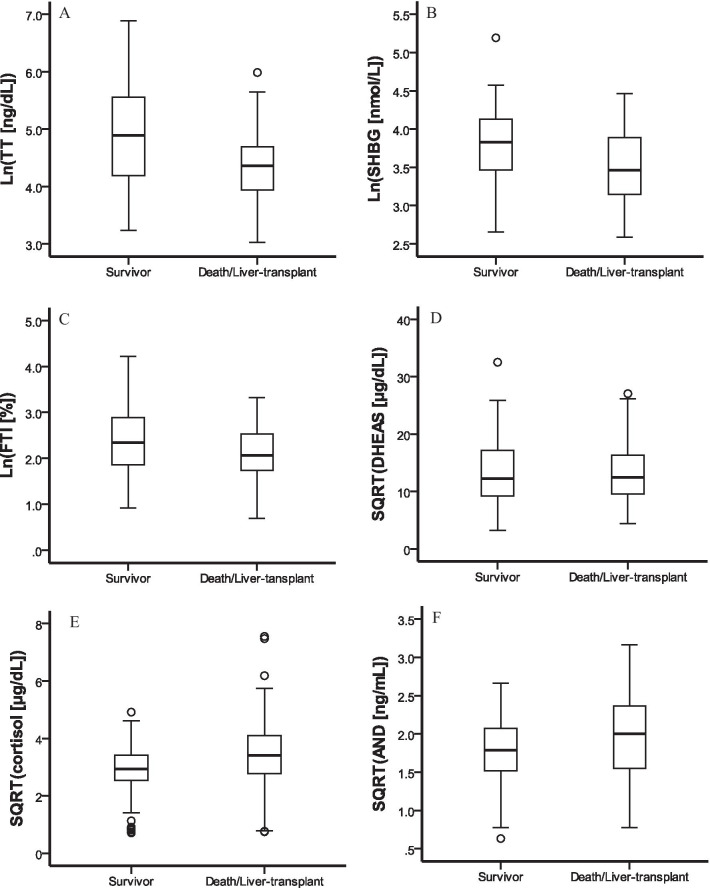
Box plot illustrating androgens in survivor and patients who experienced the composite outcome of death or transplant. The composite outcome (n = 55) was associated with lower total testosterone (*P* < 0.001, **A**), sex-hormone-binding globulin (*P* < 0.001, **B**), free testosterone index (*P* = 0.032, **C**) and higher cortisol (*P* = 0.007, **E**), androstenedione (*P* = 0.038, **F**) compared with those survived without transplant (n = 105). The dehydroepiandrosterone sulfate level was similar among those two groups (*P* = 0.686, **D**). *TT* total testosterone, *SHBG* sex-hormone-binding globulin, *FTI* free testosterone index, *DHEAS* dehydroepiandrosterone sulfate, *AND* androstenedione, *SQRT* Square Root

**Fig. 2:**
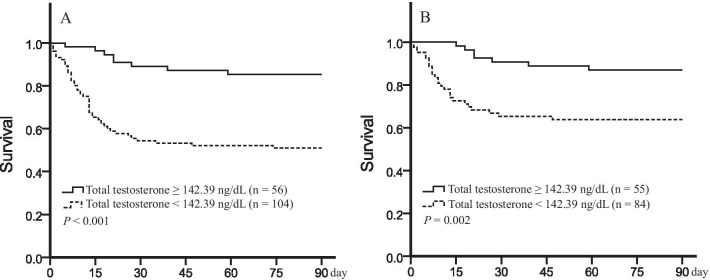
Kaplan–Meier survival curves for cases of hepatitis B virus-related acute-on-chronic liver failure. **A** Transplant-free survival without composite endpoint was lower among men with total testosterone levels below the cutoff of 142.39 ng/dL than among those with total testosterone levels above the cutoff value within 90 days. **B** Transplant-free survival without death was lower among men with total testosterone levels below the cutoff of 142.39 ng/dL than among those with total testosterone levels above the cutoff value within 90 days

After dividing patients into survivor and death/liver-transplant groups according to the 90-day outcome, we found TT, SHBG, FTI to be lower and cortisol, androstenedione higher in the death/liver-transplantation group (Fig. 1). Univariate analysis demonstrated that those patients with higher INR, urea nitrogen, Tbil, direct bilirubin, white blood cells and lower platelet count, total cholesterol and total protein had significantly greater death/liver-transplantation hazards. Cox proportional hazards analysis (Table 5) revealed low TT to be associated with an approximate quadruple risk of the composite outcome (hazard ratio: 4.49, 95% CI 2.12–9.53; *P* < 0.001) compared with normal TT. After adjustment for age, BMI, SHBG, free testosterone index, cortisol, and androstenedione (Model 2), the significant association between TT and the composite outcome remained. Model 3 showed that among the clinical variables significantly associated with the outcome in univariate analysis, only TT, INR and urea nitrogen levels were independent factors predicting composite outcome rates. As Fig. 2 illustrates, the rate of the composite outcome within 90 days was significantly higher among subjects with low TT (*P* < 0.001 by log-rank test; Fig. 2A) and low FTI group (FTI < 20.4%; *P* = 0.018). Analyzing death alone, low TT was associated with a significantly higher risk of 90-day mortality than normal TT (*P* = 0.002 by the log-rank test; Fig. 2B), while no significant differences were seen according to FTI level (*P* = 0.132).

**Table 5:** Results of Cox proportional hazards estimate of the composite outcome of death or liver transplant

Variables	Hazard ratio (95% CI)	*P* value
*Model 1*		
TT (TT < 142.39 ng/dL *vs* TT≧142.39 ng/dL)	4.49 (2.12–9.53)	< 0.001
*Model 2*		
TT (TT < 142.39 ng/dL *vs* TT≧142.39 ng/dL)	3.02 (1.33–6.86)	0.008
Age (years)		0.445
Body mass index (kg/m^2^)		0.511
1–Ln (sex-hormone-binding globulin [nmol/L])	2.04 (1.08–3.87)	0.028
1–Ln (free testosterone index [%])		0.508
SQRT (dehydroepiandrosterone sulfate [μg/dL])		0.570
SQRT (cortisol [μg/dL])	1.75 (1.32–2.31)	< 0.001
SQRT (androstenedione [ng/mL])		0.353
*Model 3*		
TT (TT < 142.39 ng/dL *vs* TT≧142.39 ng/dL)	2.57 (1.09–6.02)	0.030
Age (years)		0.837
Body mass index (kg/m^2^)		0.941
1–Ln (sex-hormone-binding globulin [nmol/L])		0.701
1–Ln (free testosterone index [%])		0.921
SQRT (cortisol [μg/dL])		0.083
SQRT (androstenedione [ng/mL])		0.068
International normalized ratio	2.32 (1.74–3.11)	< 0.001
Ln (urea nitrogen [mmol/L])	2.65 (1.82–3.85)	< 0.001
Total bilirubin (μmol/L)		0.246
Direct bilirubin (μmol/L)		0.410
Ln (white blood cell count [10^9^/L])		0.100
Ln (platelet count [10^9^/L])		0.641
Ln (total cholesterol [mmol/L])		0.343
Total protein (g/L)		0.143

Cox proportional hazards regression models for total testosterone (TT, Model 1) and models additionally adjusted for age; body mass index; sex-hormone-binding globulin; free testosterone index; cortisol; androstenedione; international normalized ratio; urea nitrogen; total bilirubin; direct bilirubin; white blood cell count; platelet count; total cholesterol and total protein. Hazard ratios are given with 95% confidence intervals within parentheses. The null hypotheses for all proportional hazard assumptions tests were not rejected for total testosterone in any model. Twenty-three patients received liver transplants and 32 died within 90 days

## Discussion

The researches on association between testosterone level and severity of HBV-ACLF in males are limited. In the present study, we confirmed that men with HBV-ACLF had lower levels of TT, FTI, DHEAS and cortisol and higher levels of androstenedione compared with age- and BMI-matched healthy controls and those with chronic hepatitis B. In secondary analysis among HBV-ACLF cases, lower levels of TT, FTI, and SHBG and higher levels of androstenedione are strongly associated with higher stages of HBV-ACLF and increased clinical severity of HBV-ACLF according to complications (ascites and hepatic encephalopathy) and multiple severity scores (MELD and COSSH-ACLF scores). Low TT is associated with an increased risk of death or the need for liver transplantation independently, but the association between FTI and this risk is weaker.

This is, to the best of our knowledge, the first prospective study to survey the associations between low serum TT and disease stage, severity, and outcome in men with HBV-ACLF. To reduce any influence of treatment, we only recruited patients who were planned to undergo treatment with the artificial liver support system, which may help to reduce the mortality rate of ACLF [15]. Our results provide insight into the role of testosterone in end stage-liver disease. Our finding of low serum TT levels in patients with HBV-ACLF is not only in line with to previous studies involving men with cirrhosis [10–12], but also true in HBV-ACLF patients without cirrhosis. When FTI is also considered, the prevalence of testosterone deficiency in HBV-ACLF seems to be more widespread. It is unlikely that our results represent an overestimation of this rate, because we collected blood samples in the morning, therefore controlling for circadian variations in testosterone levels, which are highest in the morning and lowest in the late afternoon [16]. Numerous cross-sectional and longitudinal studies have shown TT to decline with age and increasing BMI [17, 18]; however, we found the association between TT and disease stage, severity, and outcome to remain significant even after adjustment for age and BMI. Our analysis of DHEAS, cortisol and androstenedione supplements the knowledge of those androgens in liver disease and is in support of the association above.

The precise mechanism of low testosterone levels in men with HBV-ACLF is complex and involves various biological actions. Testosterone is mainly secreted by Leydig cells and regulated by hypothalamic-pituitary–testicular axis in males. Testosterone deficiency can develop as a consequence of the direct damage of the testis caused by HBV. Early in 1990, HBV DNA / RNA signals were found in testis of acute HBV infections and decompensated but not compensated CHB males [19, 20], indicating that HBV may attack testis in acute pahse instead of chronic conditions, which explains why testosterone was low in males with HBV-ACLF but normal in CHB. New evidence indicates that lipopolysaccharide, which promotes the development of ACLF [21], may initiate inflammation and result in impaired Leydig cell function thus reduce testosterone production in men [22]. Moreover, Critical illnesses, including HBV-ACLF, myocardial infarction and acute respiratory failure [23, 24], are often accompanied by dysfunction of hypothalamic-pituitary–testicular axis with hypothalamus or pituitary inhibited by estradiol and inflammation, and then testosterone secretion is downregulated. In addition, SHBG—an important determinant of the distribution of circulating testosterone—is secreted by the liver [25] and can indirectly affect testosterone levels. Researchers believed that among men infected by HIV or HCV [26, 27], TT measurement will underestimate the hypogonadism because of the “false increase” in TT induced by elevated SHBG. Likewise, in the present study, more serious liver injury was accompanied by a reduction in SHBG production (Table 1) with indirect consequences for lower testosterone levels. Finally, the abnormal conversions between androstenedione, DHEA, estradiol and testosterone caused by disordered enzymes of HBV-ACLF males may also result in testosterone deficiency.

As the most important endogenous anabolic steroid, testosterone deficiency may conceivably contribute to increased risk of advanced severity and poor outcome in patients with HBV-ACLF. A double-blind placebo-controlled trial [28] demonstrated testosterone deficiency in older men to be associated with decreased hemoglobin levels, which is supported by findings of the present study. The presence of anemia may contribute to the risk of poor outcomes; while testosterone treatment could correct anemia. Testosterone also has anabolic effects in muscle tissue, and deficiency is often accompanied by lack of exercise and poor nutrition, which is common among patients with HBV-ACLF and may lead to poor outcomes [29].

Most of all, excessive immune response triggered by HBV exacerbation is considered to be the driver of HBV-ACLF and testosterone may play a major role in it. The liver was recently demonstrated to be an androgen-sensitive organ because it expresses androgen receptors, and HBV has been suggested to be a sex-hormone-responsive virus [30]. Researchers have shown that androgen can increase HBV titer through stimulating the production of androgen response elements or via the positive feedback loop of the androgen receptor-androgen complex and HBV X protein [31, 32]. Male patients with higher levels of testosterone are prone to have higher HBV load than female patients. Once the testosterone decreased, male patients will mount a more efficient, intense, and prolonged immune response [33] which may contribute to the severity of symptoms of HBV-ACLF. In the present study, the pathophysiology of ACLF relating to excessive systemic inflammation [34] presented as an increased rate and severity of ascites and hepatic encephalopathy, which was also observed among patients with low TT and could be predicted by decreased testosterone level. Furthermore, the low-TT group of the present study exhibited elevated levels of C-reactive protein, a classic inflammatory indicator, in the absence of infection. It may be that the low testosterone levels we observed among subjects could contribute to the severity symptoms by elevating the antiviral immune defense and inflammatory responses, leading to multiple organ injury [35]. Taken together, these studies clearly demonstrate that low testosterone level is a marker of more advanced disease, although the causality in the case of HBV-ACLF remains unclear.

This study has some limitations which should be acknowledged. First, it is a single-center study which analyzed data of 160 subjects, with 55 cases of the composite outcome. Further studies involving larger cohorts recruited from multiple centers would provide information about the generalizability of these results to other populations. Second, we recruited male subjects, but changes in testosterone levels may also occur in females and affect female patients with HBV-ACLF. However, due to the rarity of HBV-ACLF in women, studies involving such subjects will require longer-term or multi-center collaborations. Finally, our observational study cannot determine the causality between low testosterone and increased death or need for transplantation among patients with HBV-ACLF. Extended longitudinal studies and prospective interventional trials may help to elucidate the underlying mechanisms of this relationship.

## Conclusions

In summary, we demonstrate the high prevalence of decreased serum testosterone levels among male patients with HBV-ACLF and show that low testosterone levels are independently associated with severity and outcomes of HBV-ACLF. Our results indicate low total testosterone level to be a marker of disease progression and poor prognosis in male patients with HBV-ACLF and testosterone supplementation may be a potential treatment to reduce the high mortality rate of male patients with HBV-ACLF, but the safety of the therapy remains to be proved in future.

## Data Availability

The datasets used and analyzed during the current study are available from the corresponding author on reasonable request.

## Abbreviations

ACLF: Acute-on-chronic liver failure
HBV: Hepatitis B virus
HBV-ACLF: Hepatitis B virus-related acute-on-chronic liver failure
BMI: Body mass index
TT: Total testosterone
FTI: Free testosterone index
SHBG: Sex hormone-binding globulin
DHEAS: Dehydroepiandrosterone sulfate
AND: Androstenedione
COSSH: Chinese Group on the Study of Severe Hepatitis B
Tbil: Total bilirubin
INR: International normalized ratio
ALT: Alanine aminotransferase
AST: Aspartate aminotransferase
MELD: Model for End-Stage liver disease
